# Tear Biomarkers in Alzheimer’s and Parkinson’s Diseases, and Multiple Sclerosis: Implications for Diagnosis (Systematic Review)

**DOI:** 10.3390/ijms231710123

**Published:** 2022-09-04

**Authors:** Angelika Król-Grzymała, Edyta Sienkiewicz-Szłapka, Ewa Fiedorowicz, Dominika Rozmus, Anna Cieślińska, Andrzej Grzybowski

**Affiliations:** 1 Faculty of Biology and Biotechnology, University of Warmia and Mazury, 10-719 Olsztyn, Poland; 2 Department of Ophthalmology, University of Warmia and Mazury, 10-719 Olsztyn, Poland; 3 Institute for Research in Ophthalmology, Foundation for Ophthalmology Development, 61-553 Poznan, Poland

**Keywords:** biomarkers, Parkinson’s disease, Alzheimer’s disease, multiple sclerosis, tears

## Abstract

Biological material is one of the most important aspects that allow for the correct diagnosis of the disease, and tears are an interesting subject of research because of the simplicity of collection, as the well as the relation to the components similar to other body fluids. In this review, biomarkers for Alzheimer’s disease (AD), Parkinson’s disease (PD), and multiple sclerosis (MS) in tears are investigated and analyzed. Records were obtained from the PubMed and Google Scholar databases in a timeline of 2015–2022. The keywords were: tear film/tear biochemistry/tear biomarkers + diseases (AD, PD, or MS). The recent original studies were analyzed, discussed, and biomarkers present in tears that can be used for the diagnosis and management of AD, PD, and MS diseases were shown. α-synTotal and α-synOligo, lactoferrin, norepinephrine, adrenaline, epinephrine, dopamine, α-2-macroglobulin, proteins involved in immune response, lipid metabolism and oxidative stress, apolipoprotein superfamily, and others were shown to be biomarkers in PD. For AD as potential biomarkers, there are: lipocalin-1, lysozyme-C, and lacritin, amyloid proteins, t-Tau, p-Tau; for MS there are: oligoclonal bands, lipids containing choline, free carnitine, acylcarnitines, and some amino acids. Information systematized in this review provides interesting data and new insight to help improve clinical outcomes for patients with neurodegenerative disorders.

## 1. Introduction

The appropriate selection of biological material is one of the most important aspects that allow for the correct diagnosis of the disease. The type, method of collection, and storage conditions have a significant impact on the reliability of the obtained result. The invasively obtained material is blood, tissue, and cerebrospinal fluid, and the non-invasive material is urine, saliva, tears, and sweat [1,2,3]. The choice of biological material depends on the possibility and usefulness for the study. A material that can be obtained non-invasively without the possibility of exposing the patient to side effects is preferred. Tears are an interesting and important biological material, due to the simplicity of collection and are related to the central nervous system (CNS) [3,4], considered an intermediate fluid between the cerebrospinal fluid and serum [5]. It was shown that proteins present in the cerebrospinal fluid were also present in tears [3]. For this reason, tears seem to be a strategic material for a new biomarkers search and for the early non-invasive diagnosis of nervous system diseases. Neurodegenerative diseases are characterized by the progressive dysfunction of the central or peripheral structures of the nervous system, and Alzheimer’s disease, Parkinson’s disease, or multiple sclerosis are examples of diseases where prompt diagnosis is very important, and biomarkers are still needed [6,7,8,9,10,11]. In this work, we reviewed the current state of knowledge, regarding the potential use of tears as an innovative non-invasive tool for the search for protein markers of nervous system diseases, such as Alzheimer’s disease, Parkinson’s disease, and multiple sclerosis. To this end, we performed an extensive search of available online databases. In this work, we relied solely on original research. Our review systematizes the available information on these diseases and marks new directions in clinical research in diseases of the nervous system.

### 1.1. Composition of Tears

Based on the Masoudi (2022) [12] and Zhou and Beuerman (2017) work [13], tear film is a composition of molecules of varied form and function of several origins. The total protein concentration of human tears ranges from 6 to 11 mg/mL, with lysozyme being the most abundant tear protein with a concentration of approximately 1 mg/mL [13]. The main components of human tears are proteins, also tear lipid, metabolites, electrolytes, vitamins, and other components [12,14].

**Figure 1:**
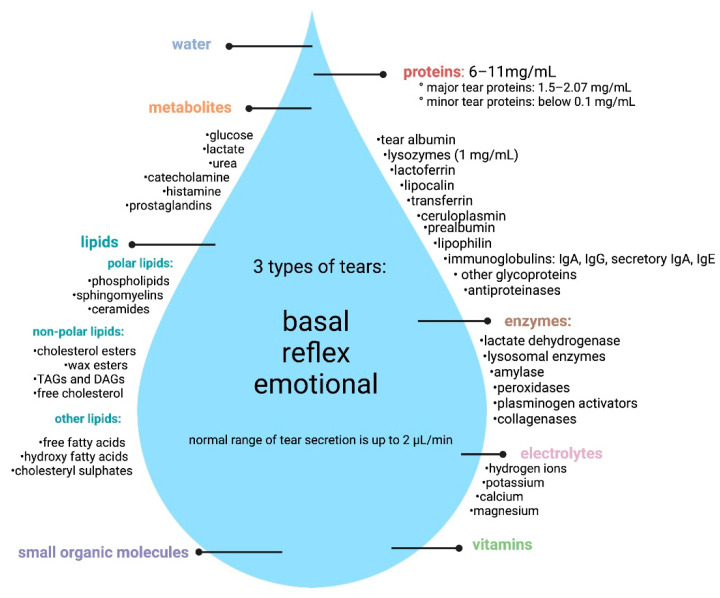
Tear composition (created with BioRender.com, accessed on 25 July 2022).

In healthy human tears, 1351 proteins were found. The principal tear proteins are directly secreted by lacrimal glands, tear lipocalin, lysosomes, and also by serum, which is probably as a result of passive transport from the blood [15]. Among the proteins detected are growth factors [16,17], cytokines [18], matrix metalloproteinases [19], immunoglobulins [20,21,22,23], sex hormones [24,25,26,27,28], proteases and protease inhibitors [29,30,31,32,33], calcium-binding proteins [34,35], and glycoproteins [36,37,38]. The tear film lipid layer (TFLL) acts as an interface between the aqueous layer and air and is the composition of different types of lipids, including cholesteryl esters (CE), wax esters (WE), and triacylglycerol (TAG) [17,39,40,41,42,43,44,45]. In tears, circulating microRNAs (cimiRNAs) as potential biomarkers were also found [46]. Wide data on other tear components are presented in the Masoudi (2022) work [12]. Figure 1 presents the main components of tears.

### 1.2. Methods of Tears Analysis

The composition of the tear fluid supplies valuable information not only about the health of the eyes but also about the functioning of the entire body. There is currently a lot of interest in the potential use of tears as a tool in health screening, disease prevention, and as a source of disease markers. Primary health care facilities where patients’ tears may be collected can play a key role. Quah et al. (2014) [47] have compared the patients’ opinion on collecting blood by antecubital venous puncture and finger prick test compared to tear collection. The analysis was performed in 383 patients (not diabetic and not attending to an eye-related complaint), whose tears were obtained using Schirmer strips. The participants also completed a pain-sensing questionnaire on a scale of 0 to 10. The authors noticed that the pain score for the collection of tears with Schirmer strips was significantly lower than for the puncture of the antecubital venous but higher than for the finger puncture. The study found that 74% of participants preferred to donate tears rather than blood for testing, confirming that tear collection with Schirmer strips is highly acceptable and can be used for primary health screening [47].

The use of the method with Schirmer strips is also appreciated by Zhou et al. [13], but these authors emphasized that it is also possible to use other absorbent materials or fire-polished microcapillary tubes. It has been shown that, regardless of the tear collection, care should be taken not to activate the corneal nerves and not induce reflex tears. Tears induced upon irritation have numerous differences in protein profile, which can influence problems and difficulties in biochemical testing.

The search for biomarkers in tears is based on obtaining biological material from patients in the control and study groups, and then laboratory analyses are carried out. Until now, a wide range of research methods have been used, including electrophoresis [48,49], spectrophotometric techniques [50], enzyme immunoassays (ELISA) [51,52], microarrays [48], and beads-based tests [53,54].

An interesting approach seems to be biosensors that are tested for example in monitoring chronic kidney disease. This non-invasive method uses the diffusion of metabolite molecules from the blood into the tears [55] and makes it possible to test not only tears but also other peripheral body fluids, such as interstitial fluid (ISF), sweat, and saliva [56]. The use of biosensors allows the monitoring of creatinine (CR), glucose, sodium, potassium, urea, and uric acid (UA), which are important indicators of homeostatic conditions. In this aspect, glucose levels can be regularly controlled, which is based on electrochemical techniques and using a special contact lens [56]. Moreover, some authors also describe the use of colorimetric methods [57]. In technological advances, microchannels have been embedded in commercial, rigid gas-permeable contact lenses for non-invasive colorimetric biodetection of the pH of tears, glucose, nitrite ions, and protein. Other authors indicated the use of an integrated tear lactate sensor using a Schirmer test strip and developed lactate oxidase, which can be used in clinical diagnostics and monitoring the performance of athletes [58]. The described tear lactate (TL) sensor has a detection range of 0.39 to 16.60 mM and is resistant to interference substances, such as acetaminophen, ascorbic acid, and uric acid. Therefore, the TL sensor seems to be a safe and painless alternative to tear measurement, and its use can be extended to dry eye syndrome, Parkinson’s disease, and cancer [58]. An interesting tool for collecting tears and glucose control is the self-diagnostic device described by Lee et al. (2019) [59]. This device was manufactured by tightly folding the tear-collecting lid in combination with a strip-type glucose sensor. The concentration in serum estimated from the tear glucose level obtained with the device showed a high correlation with the values measured with a clinically available blood glucose meter (R2 = 0.9617). The authors suggested that the use of the described device can be important in disease prevention and enables the early diagnosis of pre-diabetes [59].

The use of biosensors and contact lenses for the examination of tears has also been extensively described by other authors [60,61,62]. The tear fluid can be a valuable material for systemic glucose measurements. The method is a valuable alternative to current invasive, painful, and expensive disease monitoring methods, based on blood or serum collection.

Sempionatto et al. (2019) have proposed a more complex self-diagnostic detection system [63]. Authors described wearable tear bioelectronic platform, which is integrated with a microfluidic electrochemical detector into an eyeglasses nose-bridge pad. This system can be useful for non-invasive monitoring of key tear biomarkers, including not only glucose but also alcohol and vitamins. The device allows the collection of real-time data, and direct measurements of stimulated tears enable the creation of the first portable monitoring platform, and the principle of method is based on wireless electronics. The authors emphasized that, for the first time, a non-invasive, wearable tear alcohol biosensor mounted on eyeglasses was demonstrated. A platform located outside the eye area resolves drawbacks of the contact lens systems, including potential infections and visual disturbances [63]. The tear analysis methods are summarized in Table 1.

## 2. Methods

### Literature Search Strategy

Here, we present information on human tear biomarkers in Alzheimer’s disease, Parkinson’s disease, or multiple sclerosis, and the principal references were taken from internet databases, such as PubMed and Google Scholar, published from January 2015 until June 2022. The keywords were as follows: tear film/tear biochemistry/tear biomarkers + diseases (including Alzheimer’s disease, Parkinson’s disease, or multiple sclerosis). Google Scholar database gave different search results depending on the use of quotation marks or its absence. We included only original studies that described the biomarkers in tears. We reviewed all publications in English and those having English abstracts. 

## 3. Results and Discussion

### 3.1. Literature Search

**Figure 2:**
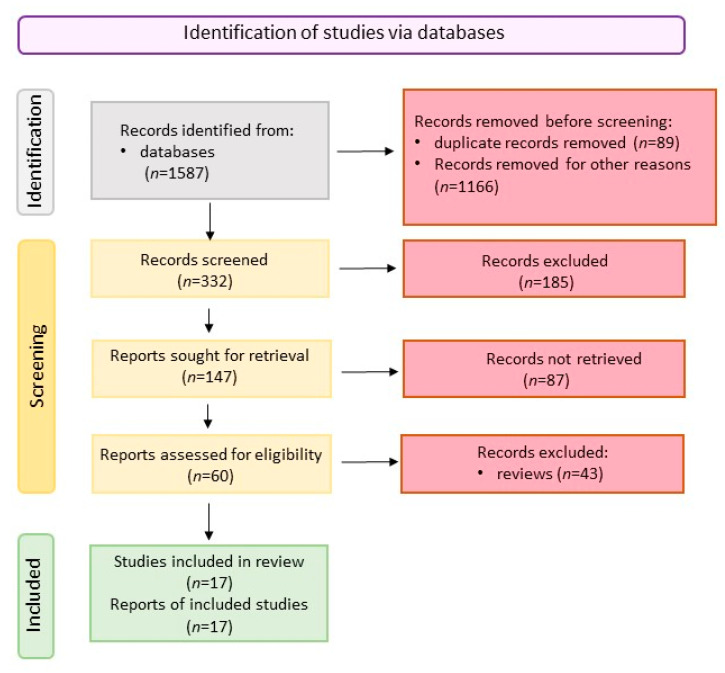
Scheme of literature searching (created with BioRender.com, accessed on 25 July 2022).

This review was performed in accordance to the PRISMA (Preferred Reporting Items for Systematic Reviews and Meta-Analyses) guidelines. Figure 2 shows the scheme of literature searching method.

### 3.2. Parkinson’s Disease Tears Biomarkers

Parkinson’s disease (PD) affects approximately 1% of the world’s population [65] and is the second most commonly diagnosed neurodegenerative disease after Alzheimer’s (AD) [66].

PD is a systemic neurodegenerative disease in which there is a loss of dopaminergic neurons in the substantia nigra of the brain and the formation of fibrous protein aggregates called Lewy bodies (LB). Until recently, PD was considered a movement disorder characterized by resting tremor, bradykinesia, postural instability, and rigidity of the limbs. Currently, in the course of PD, a multisystem disorder is observed, accompanied by inflammation of the nervous system and decreased function of the immune system. It is the cause of nonmotor symptoms, such as sleep disorders, hallucinations, gastrointestinal disorders, and scotomas [67,68,69]. The first motor symptoms appear in patients several decades after the onset of the disease process, i.e., from the onset of non-specific non-motor symptoms [70]. Changes in the human body during PD are reflected in a change in the composition of body fluids. Much research has been done to discover PD biomarkers in the blood and cerebrospinal fluid (CSF). However, none of these studies had a clear conclusion [71]. Table 2 presents the candidate biomarkers found in tears for Parkinson’s disease (PD).

Under physiological conditions, α-synuclein (α-syn) is a protein active in synapses and is involved (object) in the formation, trafficking, and coupling of synaptic vesicles; thus, α-syn is indirectly related to the recycling and storage of dopamine. The α-syn activation and deactivation process is controlled at the level of lysosomal autophagy and proteasome degradation driven by ubiquitinylation [72]. α-syn in dopaminergic neurons occurs in the form of an unfolded monomer and a folded oligomer (α-synOligo). α-synOligo is ligated and aggregated in dopaminergic neurons in the form of Lewy bodies. In the physiological state, a dynamic equilibrium is maintained between the monomeric and oligomeric forms [73]. However, in pathological conditions, α-syn undergoes non-bending folding and excessive aggregation in neurons, causing them to die [72]. Hamm-Alvarez et al. (2019) investigated the effectiveness of determining the total content of α-syn (α-synTotal) and α-synOligo in basal tears as a biomarker of early PD. The researchers also determined the concentrations of CCL2, DJ-1 protein, lactoferrin (LF), and matrix metallopeptidase 9 (MM9) in the tears of the control group (*n* = 82) and people with PD (*n* = 93) [27]. CCL2 is a chemokine used as an effector of PD progression [74]. DJ-1 is a chaperone and protects cells against oxidative stress and prevents α-syn aggregation [75]. On the contrary, LF is an indicator of lacrimal gland status [76] and MMP9 is an indicator of inflammation [77]. The researchers showed that in the tears of people with PD compared to the control group (CRT), there was a significant increase in the content of α-synOligo (CRT = 0.44; PD = 1.68 ng/mg of tear proteins) with a simultaneous reduction in the α-synTotal (CTR = 361.16; PD = 254.54 pg/mL of tear proteins). There was also an increase in total protein, CCL2, DJ-1 and MMP9, however, these changes were not statistically significant. It should also be noted that there were differences in the proteins content of the studied proteins in terms of sex between healthy people and people with PD. The level of α-synOligo in the tears of men with PD was found to increase twice, while in women as much as 4.5 times; the concentration of DJ-1 increased in men by approximately 50% and remained unchanged in women. It was also shown that in women, PD also increased the total protein content in the tears, while in men this factor remained unchanged. Additionally, only in the tears of men with PD was a statistical increase in total α-syn found. In another Hamm-Alvarez et al. (2019) study [27], they investigated reflex tear to see if it would be a more useful diagnostic tool than basal tears. It was observed that in PD patient’s tears (PD v CRT), there was a significant increase in α-syn Oligo concentration (CRT = 0.65; PD = 2.85 ng/mL of tear proteins), lactoferrin (CTR = 145.84; PD = 196.05 µg/mg of tear proteins), and CCL2 (CTR = 66.47; PD = 109.68 pg/mg of tear proteins). As in the earlier study, differences were also found between the sexes in the content of individual proteins in the tears of healthy people and the PD patient. It was shown that PD increased the content of α-synOligo in tears in men by five times and in women by four times, and the increase in CCL2 and lactoferrin during PD was found only in men. Statistical analysis of AUROC showed that α-synOligo (AUROC = 0.80) may be an effective biomarker in the diagnosis of PD. On the other hand, the combined analysis of α-synOligo and CCL2 increases the accuracy of PD diagnosis (AUROC = 0.83). Both studies also show that when looking for potential biomarkers and developing levels of diagnostic factors, it is necessary to include the gender criterion. PD is a disease that affects men 1.5–2 times more often [78]. These differences may be due to the protective role of estrogens in relation to neurons [79].

In studies conducted in the mouse model of PD, degradation of the catecholaminergic systems was demonstrated [80]. The degradation of central and peripheral catecholaminergic neurons during PD affects changes in the motor functions of the muscles and the eye itself [81]. Therefore, it seems justified to study the change in the profile of monoamine accumulation in tears as a potential source of PD biomarkers. Bogdanov et al. (2021) [82] analyzed the change in the concentration of selected catecholamines (dopamine, noradrenaline, adrenaline), metabolites: L-DOPA (dopamine precursor) and DOPAC (dopamine degradation product). The study also analyzed the content of α-2-macroglobulin, which is a proteases inhibitor present in patients’ PD tears and involved in the pathogenesis of PD [82]. The change in the regulation of this protein is observed in the CSF of people with PD [83]. This study included 31 people with early stage PD and 32 people in the control group. From the data collected, researchers showed that PD develops asymmetrically. The following pattern of changes in the content of tested proteins in PD tears, compared to the control group was found: α-2-macroglobulin on both sides and a 2-fold reduction in adrenaline content regardless of the side of the eye. The authors also performed a statistical analysis of the sensitivity and specificity of the parameters tested. It shows that adrenaline, noradrenaline, and the analysis of α-2-macroglobulin activity have the greatest potential as biomarkers (81.2%, 88.9%, and 92%, respectively). α-2-macroglobulin shows a large variety of functions; it is difficult to clearly define its increased activity in the tears of PD patients. The protein exhibits neuroprotective activity by stabilizing misfolded proteins (α-synuclein and β-amyloid), preventing their aggregation and transformation into neurotoxins [83]. On the other hand, α-2macroglobulin, due to the fact that it inhibits the activity of the neuronal growth factor, shows a neurotoxic effect [84]. Regardless of the function of α-2-macroglobulin, it has a high potential to be a biomarker of the preclinical and clinical stage of PD. Another study also looked at the change in catecholamine content in tears in PD subjects [85]. It was found that in PD patient’s tears, compared to the control group, the concentration of norepinephrine increased twice, the dopamine content increased by about 50% but only after the ipsilateral site, and the concentration of epinephrine on both sides decreased twice. There is a risk that the detected biomarker in patients in the clinical stage is absent in the preclinical stage. However, Kim et al. (2019*) [85] are convinced that the catecholamines identified in the study have a high potential to be markers of the early and clinical stage of PD. The thesis of these authors may be confirmed by the results of studies on the animal model of PD, where an increase in the content of norepinephrine in tears was also observed in the preclinical phase [86] by Kim et al. (2019**).

Proteomic analysis using LC-MS provides a wealth of key data to understand the molecular basis of systemic neurodegenerative diseases. This type of analysis is also the best tool for the precise search for whole groups of biomarkers specific for a given disease entity. This research strategy was used by Boerger et al. [87]. They performed the segregation and tear identification of 36 PD patients and 18 controls. A total of 571 were identified in tears PD and CRT. Only 31 proteins were present in the tears of PD patients and only 7 in the tears of healthy subjects. It was also shown that 21 proteins increased and 19 decreased in the tears of PD patients, compared to the control group. The performed gene ontology (GO) analysis allowed the determination of the functions of proteins, the accumulation of which changed in tears during PD. A total of 40% of these proteins were related to neuronal function. Seven proteins were associated with the maintenance of retinal homeostasis and eight with the myelin sheath. Of the proteins present only in PD tears or proteins that were up-regulated during PD, 40% were associated with lipid metabolism and 10% with oxidative stress. Additionally, protein–protein interaction (PPI) analysis revealed many interactions between proteins related to vesicle-mediated transport, secretion, stress response, and wound healing. The results obtained may indicate a significant influence of these proteins on the pathogenesis of PD. Furthermore, the authors in their study found the following pattern of protein accumulation: protein deglycase DJ-1 [PARK7] was recovered from CTR tears and with numerically higher levels in PD tear samples, S100 superfamily (i.e., [S100A7], [S100A8] and [S100A11]), Peroxiredoxin-6 [PRDX6], Annexin-A5 [ANXA5], and Glutathione S-transferase-A1 [GSTA1] that were upregulated in the PD group. Several proteins from the apolipoprotein superfamily (i.e., [ApoD], [ApoA4], and [ApoA1]) were increased in tears of PD patients. However, the authors do not indicate unequivocally which of the proteins identified by them is a PD biomarker. Among the potential candidates, they mention apolipoprotein D, which is involved in cholesterol binding and transport, and its increased amount has been detected in the black essence of patient PD [88]. The authors also point to the potential of the identified serum paraoxanse/arylesterase 1 (PON1) protein related to organophosphate metabolism. Polymorphism in PON1 is associated with an increased risk of PD [89]. Lactotransferin, clusterin, and beta-2-microglobulin were indicated in the group of immune response-related proteins with a high prognostic value. The authors note that these were a pilot study and that the analyses should be repeated in a larger population to validate them [82]. A valuable supplement to the knowledge about the PD substrate are the results of Acer et al. (2022) [17], where authors are already jumping on protein candidates in the pre-clinical phase of PD. In this study, there were 3 people in the PD study group (*n* = 24) with the E46K mutation in α-syn (E46K-SNCA). Mutations and multiplications in the SNAC gene are associated with an increased risk of autosomal dominant familial PD. On the contrary, the most aggressive form of PD is associated with the E46K-SNCA mutation [90]. A total of 560 proteins have been identified in the CTR and PD tears. Proteins that were deregulated in the PD tears of the patients were mainly associated with immune response, apoptosis, collagen degradation, protein synthesis, lipid transport, and defense. The accumulation pattern of PD patient proteins with the E46K-SNCA mutation differed from the PD patient without this mutation. However, an increase in the accumulation of the same proteins was also found in both forms of PD, while in the PD patient with the mutation, they were more strongly expressed. The authors distinguished a group of six proteins that were subject to variable regulation in PD patients’ tears, five up-regulated: preamine AIC (LMNA) (fold change 2.25), cathepsin D (CATD) (fold change 1.85), acid ceramidase (ASAH1) (fold change 1.8), transitional endoplasmic reticulum ATP-ase (TERA) (fold change 1.6), and cytoplasmic dynein-1 heavy chain (DYHC1) (fold change 1.32) and one down regulated: tripeptidyl-peptidase 1 (TPP1) (fold change 0.64). These proteins are associated with neurodegeneration processes, such as apoptosis, lysosomal autophagy, demyelination, and axonal transport. The statistical analysis (ROC) showed that three of the six of these proteins show a high ability to classify patients with PD. They are: CATD (AUC = 75.1%), ASAH1 (AUC = 7.3%), and DYHC1 (AUC = 70.2). It should be emphasized that this study also found a relationship between these proteins and age. CATD and ASAH1 are proteins associated with lysosomal autophagy. CATD is a lysosomal protease involved in α-syn metabolism. ASAH1 is a lysosomal enzyme that converts ceramide to sphingosine. It has been shown that increased ASAH1 activity causes a decrease in ceramide content, which leads to excessive intracellular α-syn accumulation [91]. In contrast, DYHC1 is a cytoplasmic protein associated with the control of the movement of cell organelles and retrograde transport in axons [92].

**Table 2:** Characteristic of biomarkers in Parkinson’s disease.

Biomarker(s)	Number of Patients	Method of Tears Collection	Method of Identification/Analysis	Results	References
Oligomeric α-synunuklein (α-synOligo); total α-synuklein (α-synTotal);	CTR *n* = 84; PD *n* = 94	Schirmer strip (*basal tears*)	ELISA	**(1)** α-synTotal decreased significantly in PD, compared to CTR (*p* = 0.004); **(2)** α-synOligo increased significantly in PD compared to CTR (*p* = 0.001); **(3)** the level of changes in analyzed parameters was associated with sex; **(4)** total protein, CCL2, DJ-1, and MMP9 were increased in PD but changes were not statistically significant.	[93]
Oligomeric α-synunuklein (α-synOligo); total α-synuklein (α-synTotal) CCL2; lactoferrin (LF)	CTR *n* = 82; PD *n* = 93	Schirmer strip (*relax tears*)	ELISA	**(1)** α-synTotal decreased significantly in PD, compared to CTR (*p*-value = 0.05); **(2)** α-synOligo, lactoferrin, and CCL2 increased significantly in PD, compared to CTR (respectively: *p* = 0.005; *p* = 0.002; *p* = 0.003); **(3)** Level of changes analyzed parameters were associated with sex; **(4)** AUROC test for α-synOligo was 0.80 and for α-synOligo and CLL2 was 0.83.	[27]
Norepinephrine; adrenaline; α-2-macroglobulin	CTR *n* = 32, PD *n* = 31	Schirmer strip	HPLC	**(1)** Noradrenaline increased in PD mainly on the ipsilateral side of pronounced motor symptoms (72%, *p* = 0.049); **(2)** a decrease in adrenaline level on both sides (ipsilateral—53%, *p* = 0.004; contralateral 42%, *p* = 0.02); **(3)** increased α-2-macroglobulin activity on both sides (ipsilateral 53%, *p* = 0.03; contralateral—56%, *p* = 0.037), compared to CTR; **(4)** adrenaline, noradrenaline, and the analysis of α-2-macroglobulin activity have the greatest potential as a biomarker (81.2%, 88.9% and 92%, respectively).	[82]
Epinephrine; norepinephrine; dopamine	CTR *n* = no data, PD *n* = 26	Schirmer strip	HPLC	**(1)** In PD, the concentration of norepinephrine increased twice; **(2)** the dopamine content increased by approximately 50% (only the epinephrine ipsilateral site), and the concentration on both sides decreased twice.	[85]
Protein deglycase DJ-1 [PARK7]; S100 superfamily; peroxiredoxin-6 [PRDX6]; annexin-A5 [ANXA5]; glutathione S-transferase-A1 [GSTA1]; apolipoprotein superfamily	CRT *n* = 18, PD *n* = 36	Schirmer strip	LC-MS/MS	**(1)** 571 proteins were identified in PD and CTR; 31 proteins were exclusively detected in the PD and only 7 in the CTR group; **(2)** 21 proteins were significantly increased and 19 decreased in the PD versus CTR; **(3)** Proteins involved in immune response, lipid metabolism, and oxidative stress were distinctly altered in PD; **(4)** Protein deglycase DJ-1 [PARK7], S100 superfamily (i.e., [S100A7], [S100A8] and [S100A11]), peroxiredoxin-6 [PRDX6], annexin-A5 [ANXA5], and glutathione S-transferase-A1 [GSTA1] were upregulated in the PD; **(5)** Several proteins from the apolipoprotein superfamily (i.e., [ApoD], [ApoA4] and [ApoA1]) were increased in tears of PD.	[87]
cathepsin D (CATD); acid ceramidase (ASAH1); cytoplasmic dynein-1 heavy chain (DYHC1)	CTR *n* = 27, PD *n* = 24, PD (*with E46K-SNCA mutation*) *n* = 3	Capillaries (*glass*, 10 µL)	nLC-MS/MS	**(1)** 560 proteins have been identified in CRT and PD tears; **(2)** Proteins deregulated in the PD were mainly associated with immune response, apoptosis, collagen degradation, protein synthesis, lipid transport, and defense; **(3)** The group of 6 proteins that were up-regulated was distinguished: preamine AIC (LMNA), cathepsin D (CATD), acid ceramidase (ASAH1), transitional endoplasmic reticulum ATP-ase (TERA), and cytoplasmic dynein-1 heavy chain (DYHC1) and one down-regulated: tripeptidyl-peptidase 1 (TPP1) in PD; **(4)** tree proteins showed a high ability to classify patients with PD: CATD, ASAH1, and DYHC1.	[17]

CTR—control group; ELISA—enzyme-linked immunosorbent assay; HPLC—high-performance liquid chromatography; LC-MS/MS—liquid chromatography with tandem mass spectrometry; nLC MS/MS—nano liquid chromatography with tandem mass spectrometry; PD—Parkinson’s disease.

### 3.3. Alzheimer’s Disease Tears Biomarkers

Alzheimer’s disease (AD) is the most common cause of dementia (70% of cases), the seventh leading cause of mortality globally, one of those diseases with the highest cost to society, and always fatal. Nowadays, AD cases are estimated at more than 55 million worldwide, with forecasts reaching 78 million by 2030. At the same time, the data indicate also that probably less than 25% of cases globally are actually diagnosed, and in lower-income countries recognizability percentage may be as low as 10%. Since more than half of patients are over 85 years, this disease has the highest prevalence among older adults (8% in the population above 85). However, it is believed that it starts much earlier, even 20 years before the first clinical symptoms, i.e., around the fourth decade of human life [94,95]. AD is classified as a neurodegenerative disorder caused by the loss of neurons in the brain, particularly the cortex, which leads to progressive cognitive, behavioral, and motor impairment and ultimately death. The disease process is associated with the accumulation of senile plaques (deposits of amyloid-beta) and neurofibrillary tangles (NFT, deposits of tau protein) in the brain. An AD-affected brain shows atrophy of cerebral cortex responsible for language and processing information, atrophy of hippocampus responsible for the formation of new memories, and expanded cerebral ventricles. Other pathogenic mechanisms of AD, overlapping with or induced by Aβ plaques and NFT were also indicated, such as inflammation, oxidative damage, iron and cholesterol metabolism alternations, blood–brain barrier (BBB) dysfunction, or α-synuclein toxicity [94,96]. It is worth pointing out, that although AD is usually defined as a strictly homogeneous CNS disease it seems that there are reasons that could allow its classification as a systemic disease. The hypothesis has been proposed in 2014 by American scientists and emphasizes the role of invalid lipid metabolism, calcium homeostasis disturbances, and mitochondrial dysfunctions in the disease progress. These biochemical disturbances are related to CNS dysfunctions of CNS and the systemic processes, such as body composition and nutritional status, general physical condition, or muscle and bones status. Symptoms, such as these, could be observed as discrete, non-specific changes in the prodromal stage of AD or even in its preclinical phase [97]. Structural and functional changes in the visual system of AD patients were also reported. Quite common are visual deficits, such as loss of visual field, decreased contrast sensitivity, low visual acuity, impaired color vision or motion perception, and visuospatial deficits [98]. In symptomatic AD, the nerve cell layer thinning, optic nerve atrophy, loss of retinal ganglion cells, and changes in retinal vasculature could be found [99]. Moreover, APP and AD-related peptides metabolism in the cornea, as well as Aβ plaques and NFT presence in the retina and lens have been detected, wherein the appearance of misfolded proteins deposits in the AD patient’s eyes were correlated with the cerebral aggregates [99,100]. Patients with AD can express reduced corneal sensitivity and disturbances in tear functionality due to their incorrect spreading on the ocular surface or drainage, which is expected to be related to pathological cholinergic transmission, the other hallmark of AD [7,101]. Few AD cases (about 5%) are classified as early onset familial Alzheimer’s disease (EOAD), the condition characterized by the onset of dementia at less than 65 years old, a positive familial history of dementia, possible mutations in presenilin (PS1, PS2) or amyloid precursor protein (APP) genes, and a more aggressive course [102]. Most AD cases (95%) diagnosed above 65 years (late-onset sporadic Alzheimer’s disease, LOAD, or AD) are considered idiopathic at this moment. The diagnosis is usually elusive until a later stage, as the disease progresses slowly for an exceptionally long time and is initially asymptomatic. The average survival time from diagnosis is approximately 10 years, of which almost half is associated with loss of independence by the patient [103].

For many years, the diagnosis of AD was based on an analysis of the common symptoms, the order in which they occur, and the rate of progression. The databases were standardized using neuropsychological tests, family interviews, and differential diagnosis, excluding other causes of dementia. It is estimated that such an approach resulted in diagnostic sensitivity and specificity of about 81% and 70%. In addition, because it was based on clinical symptoms, it allowed for diagnosis at a relatively advanced stage of the disease. Additionally, the final certainty could only be obtained by brain biopsy with histopathological methods, usually after an autopsy. With the progression in understanding the biological basis of the disease, as well as with the considerable progress in the diagnostic methods of AD that has come with the development of imaging techniques and omics technologies, the diagnostic criteria for AD have been revised by different authorities. Although the DSM-5 and ICD-11 recommendations still focus solely on clinical diagnosis, the NINCDS-ADRDA (National Institute of Neurological and Communicative Disorders and Stroke with the Alzheimer’s Disease and Related Disorders Association) and NIA-AA (National Institute on Aging with the Alzheimer Association) recommendations were more profound. Two significant changes were made. Firstly, the disease starts to be considered as a spectrum that could be diagnosed in one of three stages: preclinical (no symptoms), mild cognitive impairment (MCI) or prodromal AD, and Alzheimer’s dementia. Secondly, biomarker analysis as additional tests supporting early diagnosis has been introduced. All biomarkers were classified with the ATN system in three groups: (A) β-amyloid deposition biomarkers (Aβ42 or Aβ42/Aβ40 ratio in CSF, amyloid-PET), (T) pathologic tau protein biomarkers (p-tau in CSF, protein tau-PET), and (N) neurodegeneration biomarkers (total tau protein in CSF, reduced glucose metabolism in bilateral temporal-parietal regions in FDG-PET, and atrophic changes in the medial temporal lobe in MRI). It was emphasized, however, that preclinical AD is an experimental concept only, and biomarkers, although strongly indicating a preclinical phase, still do not predict which cognitively healthy individuals will progress to MCI or dementia. On the other hand, the imaging techniques in tandem with molecular biomarkers could not only move the time of diagnosis to the asymptomatic period (preclinical AD) but also increase diagnosis sensitivity and specificity for MCI or AD dementia stage up to 92% and 90%, respectively [94,104,105,106].

The causal pharmacological treatment for AD is still not available. In fact, the first-ever drug for AD (Aducanumab), a monoclonal antibody that removes beta-amyloid deposits from the brains of early stage patients, had just received FDA accelerated approval (June 2021), and was pending approval in Europe [107]. Meanwhile, AD is being treated with drugs that improve cognitive functions (cholinesterase inhibitors and memantine) and non-pharmacological management of the cognitive and behavioral symptoms are used. The fact is that the progression of the disease (neurodegeneration) cannot be stopped or reversed, even by Aducanumab. However, it can be slowed down. That emphasizes why early and precise diagnosis is so important in AD [96,103].

The early diagnostic biomarker must be specific, sensitive, reproducible, non-invasive in the collection, inexpensive to measure, and easy to implement on a larger scale (standardizable). As genetic risk markers (PS-1, PS-2, APP gene mutations and APOE4 allele variant) have no strong diagnostic or prognostic value; they are not recommended in routine clinical practice in early AD diagnosis. Imaging techniques are not commonly available and quite expensive; hence, in practice they are less frequently used but are still used to confirm or exclude AD in an uncertain diagnosis. Therefore, great emphasis is placed on the search for markers that are soluble in body fluids [95].

The biological material of the first choice for searching of markers in neurodegenerative diseases is CSF. As a draining interstitial fluid, it is in the closest proximity to the CNS cells and can thus best reflect pathological processes occurring during Alzheimer’s disease. Additionally, it has the potential for a high concentration of candidate biomarker(s). Up to today, except for the presence of two classical AD markers (Aβ peptides and p-Tau), about 16 other proteins involved in the pathological processing of APP, neuroinflammation, and synaptic dysfunction, a similar number of down- or up-regulated miRNA, and several types of lipids have been proposed as biomarkers of AD. Unfortunately, its collection is quite invasive, carries a certain risk of harm to the patient, and requires well-trained clinicians. The procedures associated with can be painful and usually cause serious anxiety in patients [100].

From a clinical point of view, the best source of biomarkers is blood, that is less expensive than neuroimaging and less invasive than CSF. As the material it is relatively easily available, but, on the other hand, it is also a rather complex matrix. Additionally, in the case of neurodegenerative disorders markers, the functioning of the blood–brain barrier should be taken into consideration. Thus, the number and the quantity of CNS-derived biomarkers are rather low in the blood, and they may be subjected to interference during the analyses. Both aspects could affect less consistent results and make the research of neurodegenerative biomarkers in blood challenging. However, blood analyses are still of great interest to clinicians; therefore, efforts to use this biological material are still being made. Promising blood markers for AD are: amyloid Aβ 42/40 (amyloidogenesis), p-tau analytes (tau pathology), neurofilament light chain (NfL), and neutrophin-1 precursor (NT1) (neurodegeneration), as well as glial fibrillary acid protein (GFAP) (astrocytic activation) [95,100]. Noteworthy is the interesting way “how to sample components of brain tissue non-invasively”—in the blood, which has opened with the discovery of new exosomes functions. CNS-derived exosomes apart from different physiological roles, serve also as supportive disposal machinery of accumulating, unwanted biomolecules. They were found to play a crucial role in of progression the neurodegenerative diseases, e.g., in the cell-to-cell spread of amyloidogenic proteins. As exosomes can cross BBB, it makes them a highly attractive source of biomarkers originating in the CNS that could be isolated from the blood. A particular advantage of biomarker analysis in CNS-derived blood exosomes is the ability to compare biomarkers in exosomes originating in different cell types (neurons, neural precursor cells, and astrocytes). Multiple protein and miRNA markers were analyzed, and some could be potential markers in AD but more studies are needed to confirm their usefulness [100,108].

The close anatomical and functional relationship between the eye and the brain means an especially interesting alternative source of biomarkers for AD might be the patient’s tears. As an easily accessible body fluid, with a relatively simple and stable composition and non-invasive sample collection tears could be a biological material for searching for AD markers that combine the favorable features of both previous ones—CSF and blood. Furthermore, the finding of AD early markers in tears could be especially valuable for the screening of general populations [99]. To date, five different studies have been conducted to find AD biomarkers in the tear’s fluid. They were concerned with qualitative and quantitative determinations of classical AD biomarkers, as well as possible new biomarkers of the disease with several types of immunoassays, proteomic, or molecular biology analyses (Table 3).

The two classic types of biomarkers for AD are Aβ peptides and different forms of Tau protein. The metabolism of APP protein produces several amyloidogenic peptides 37–43 amino acids long, where the most abundant in the body are Aβ40 and Aβ42. The solubility of amyloidogenic peptides is low, and under physiological conditions in blood or CSF their concentrations fluctuate in the range of 4–400 pg/mL. It was found that the concentration of Aβ40 is usually higher than that of Aβ42 (e.g., in CSF Aβ40 constitutes up to 70% and Aβ42 about 10%). At the same time, Aβ42 is more amyloidogenic, shows greater cytotoxicity, forms fibrils faster, and is the main component of senile plaques in AD. Therefore, it is considered to be more directly related to AD dementia. However, many studies indicate that more relevant to AD pathogenesis is not the absolute concentration of Aβ42, but the ratio Aβ42/Aβ40 [109]. Another hallmark of AD is tauopathy, although it can also be found in other diseases of the CNS. Tau is a neuronal protein stabilizing microtubules that has a large number of phosphorylation sites. Hyperphosphorylation of tau constitutes an important molecular abnormality of Alzheimer’s disease. The total concentration of Tau protein (t-Tau) in the blood or cerebrospinal fluid is used as an indicator of neurodegeneration (CSF t-Tau concentrations in Alzheimer’s disease might reach even 300% of control levels), while its phosphorylated form at Thr_181_ (p-Tau181) is indicated as a typical marker of Alzheimer’s disease in CSF [95].

The presence of Aβ42 in the tear fluid was confirmed by the three independent research teams. Del Prete et al. (2021) [110] found this peptide by immunocytochemistry assay in tear smears of two people at family risk of AD, and at the same time showed its absence in tears of a healthy person. Moreover, the presence of Aβ42 in tears was linked with retinal plaques in these subjects. Since all donors were cognitively normal, the authors suggested the possible predictive value of tears Aβ42 occurrence in the diagnosis of AD. The existence of both peptides (Aβ40 and 42) in the tears of healthy people aged 20 to 79 was also demonstrated by Wang et al. (2021) [111] who, using a new type of electrochemical immunosensor, found that the amount of both peptides could be even 10 times higher in the tear fluid (10 pg/mL level) than in whole blood (1 pg/mL level) and that Aβ concentrations in the different age groups could be related to a certain degree with the age. More extensive research of classical AD biomarkers in the tears of patients with different degrees of cognitive impairment (subjective cognitive decline—SCD, mild cognitive impairment—MCI, and Alzheimer’s dementia—AD) was carried out by Gijs et al. (2021) [112]. Due to the use of the multiple immunoassay platform, they determined five different parameters in the samples in terms of the pathology of amyloid proteins (triplet assay for Aβ-38, -40, and -42) and Tau protein (duplex assay for t-Tau and p-Tau). They found that the detectability of amyloid peptides in tears was high only for Aβ40 type. The remaining two were present in less than 23% of all samples, with Aβ42 being determined mainly in the healthy group. The measured levels of amyloid peptides were higher in the three groups of patients (median 17 to 1680 pg/mL) than in the healthy group (median 4 to 60 pg/mL), but the differences were not significant. This also did not differentiate the groups of patients. Unfortunately, due to the low detectability of Aβ42, the ratio Aβ42/Aβ40 could not be estimated. Similarly, they had inconclusive results with the Tau protein. They found a high detectability of total Tau protein in the tear’s fluid (94% of samples) with concentrations at the ng/mL level, i.e., about 10 times higher than the levels measured in the CSF. Unfortunately, although higher concentrations of Tau protein were found in patients with cognitive impairment than in healthy ones, these differences were not significant. However, tears t-Tau levels were significantly higher in a subgroup of patients with increased neurodegeneration parameters in CSN (N-positive) compared to N-negative patients. Lastly, the phosphorylated form of the Tau protein, such as Aβ42, before, had an exceptionally low detectability in tear samples (18%). Nevertheless, it should be noted that p-Tau was identified only in the study groups but not in the control group. So, taking all the above into account, it can be said that classical AD biomarkers are possible to be measured in the tear’s fluid, with levels appearing to be higher than in other biological materials (blood, CSF). However, whether they have predictive value for Alzheimer’s disease and whether they will become diagnostically useful requires further research. From the laboratory practice side, a significant hindrance here is the small volume of the tears sample and the need of its dilution for the determination of the analytes. Such a preparation, in the term of marker quantification, requires the latter use of appropriate conversion factors, including the sample volume and the dilution factor. Standardization of the first parameter could be particularly challenging.

A new type of biological material also opens up the possibility of searching for completely new biomarkers of disease. So far, two other teams have taken this problem in the diagnosis of the Alzheimer’s disease area with the use of tears fluid. Kenny et al. (2019) [113] have analyzed the complete tears proteome of AD, MCI, and healthy patients and made comprehensive analyses of the protein profiles in combination with the bioinformatic GO analysis. Although researchers did not find a clear pattern of a disturbed biological process characteristic of AD, they identified 12 such proteins that were specific to dementia patients. These proteins fell into the category related to the regulation of endopeptidase activity, protein folding, regulation of cellular amino acid metabolism, and regulation of mRNA stability. In their study, the most relevant protein, as present only in the AD group, was eukaryotic translation initiation factor 4E (eIF4E). Unfortunately, the researchers failed to confirm the presence of this protein on Western blot. Thus, they concluded that in addition to the individual variability of tears proteins, possible contamination by patient’s cells could also contribute to the inconsistent protein detection in the samples. This remark might be useful for optimizing tear fluid sampling in other studies. Another result of the team was the identification of seven unique proteins found only in the MCI patients, but none of them were identified with the frequency appropriate for a potential marker (in more than 50% of samples). However, an interesting observation was that most of the twelve AD-specific proteins showed increased expression in the MCI group compared to the control samples, suggesting an incremental change in disease progression. The researchers of the second team, Kallo et al. (2016) [101], were focused on a detailed proteomic analysis solely in the area of differences in the global changes in the protein profile of tears between AD patients and healthy people. As a result of SDS-PAGE and subsequent LC-MS/MS analyses, they identified 10 proteins with markedly different intensities between the groups. In the next SRM-based targeted MS analyses, they showed that in tears from AD patients, five of them are present in significantly lower amounts, whereas one is significantly more highly expressed. Finally, the ROC analysis showed that the best potential marker for AD, with an 81% sensitivity and 77% specificity, is the use of a combination of four proteins: lacritin, lipocalin-1, lysozyme-C, and dermcidin. Since the first three listed are expressed mainly by the lacrimal glands and are clearly down-regulated in AD patients, they also concluded that lacrimal gland dysfunctions could be possible in addition to the processes of neurodegeneration in Alzheimer’s disease.

Recently the emerging types of disease biomarkers are miRNAs. These small, noncoding, and stable types of exogenous RNA can be found in different body fluids, among others, in the peripheral blood, CSF, and tears. The blood and CSF miRNAs were also tested for their use in the diagnosis of AD. For example, a panel of 10 miRNAs in the blood has been hypothesized to be deregulated early in Alzheimer’s disease, before the onset of clinical symptoms. These RNA markers were found to be associated with the immune system, cell cycle, gene expression, cellular response to stress, neuron growth factor signaling, Wnt signaling, cellular senescence, and Rho GTPases [114]. Several putative microRNA biomarkers have also been identified in the CSF, but their utility is disputable by the practicality of its invasive collection method. Tear fluid is not a common material in these types of analyses, although it has been used in research on ophthalmic diseases in recent years. In contrast, the analysis of the microRNA profile in the tears of patients with cognitive impairment so far was carried out only by Kenny et al. (2019) [113]. The first thing they found out was a significantly higher concentration of small RNA levels in AD, compared to MCI and control. The exact cause of the condition has not been established, but the authors suggest its relation to the presence of neurodegenerative and neuroinflammatory processes in AD. Secondly, as a result of the miRNA profiling performed with a genome-wide high-throughput qPCR-based microRNA platform they found a set of small RNAs specific only for tears fluid of MCI and AD patients, with mi-RNA-200b-5p occurring only in patients with dementia. Unfortunately, its relation to the pathological processes of Alzheimer’s disease is currently unknown. However, it should be noted that due to the limited specimen size these analyses were performed on pooled samples. Hence, as suggested by the authors, future studies with a greater sample size should be executed to replicate and extend these findings.

**Table 3:** Characteristic of biomarkers in Alzheimer’s disease.

Biomarker/s	Number of Patent	Method of Tears Collection	Method of Identification/Analysis	Results	References
Lipocalin-1; dermcidin; lysozyme-C: lacritin;	CTR *n* = 9, AD *n* = 14	Capillaries	LC-MS/MS; SRM-based targeted MS *(with ROC analysis)*	**(1)** Tear flow rates were significantly higher in AD (12 ± 2 μL/min) than in CTR (6 ± 2 μL/min); **(2)** Total protein concentration in tears was significantly higher in AD (8.8 ± 2.9 μg/μL) than in CTR (4.4 ± 1.4 μg/μL); **(3)** 10 proteins presented a significantly different intensity in AD group, compared to CTR; **(4)** The ROC analysis of the quantitative SRM-based proteomic results demonstrated the combination of 4 proteins that could be a potential biomarker of AD (with 81% sensitivity and 77% specificity); **(5)** Lacrimal gland dysfunction could be possible in AD as three of biomarker proteins: lipocalin-1, lysozyme-C, and lacritin (all typical of the lacrimal glands) were found to be downregulated in AD group.	[101]
eIF4E (and others 11 proteins); 38 miRNAs (mainly miRNA-200b-5)	CTR *n* = 15, MCI *n* = 8, AD *n* = 9	Schirmer strips	high throughput RP-LC-MS/MS; genome-wide high-throughput qPCR-based microRNA platform (*OpenArray*)	**(1)** Tears flow rates were not significantly different between groups (Control (12 ± 9); MCI (9.25 ± 6.3) and AD (8.5 ± 2.9) estimated in mm/5min); **(2)** Total proteins concentration in tears was similar between all groups; **(3)** Profiling of proteins in a complete tears fluid proteome analysis did not show the presence of classical markers for AD; **(4)** GO analysis did not show a clear pattern in AD patients, but typical AD protein categories were found to contribute to the regulation of endopeptidases activity, proteins folding, cellular amino acid metabolic processes, and mRNA stability; **(5)** A unique proteomic and microRNA composition may be present in tears fluid of AD patients—12 AD-specific proteins were identified (with eIF4E presented only in AD group) and 38 distinct microRNAs (with miRNA-200b-5p detected in AD samples only).	[113]
Aβ38, Aβ40, Aβ42, t-Tau, p-Tau	CTR *n* = 9, SCD *n* = 23, MCI *n* = 22, AD *n* = 11	Schirmer strips	Multiplex immunoassays with electrochemiluminescence	**(1)** Tear fluid classical AD biomarkers detectability: Aβ40 and t-Tau were detectable in more than 94% of samples, while Aβ38, Aβ42, and p-Tau were detectable in less than 23% of samples; p-Tau was not detectable in the TR; Aβ42 was better detectable in CTR (78%), as compared to all patients (<18%); **(2)** Levels of classic AD biomarkers levels: detection level for amyloid peptides was pg/mL and for Tau protein forms was ng/mL (10 times higher than in CSF); Aβ38, Aβ40, Aβ42, and t-Tau were higher in patients, compared to CTR (but not significantly); p-Tau did not differ between patient groups; **(3)** Levels of biomarkers in tears of patients classified according to the ATN criteria: t-Tau levels were significantly elevated in patients with neurodegeneration (N positive), compared to patients without neurodegeneration (N negative).	[112]
Aβ42	CTR *n* = 1 *(healthy, no family risk)*, AD *n* = 2 *(healthy, with family risk),*	No data	Immunocytochemistry assay *(and fundus camera examination for retinal plaques)*	**(1)** Numerous plaques on the retina were found in patients with a family risk of AD only; **(2)** Aβ-42 peptide was present in tears of patients with a family risk of AD only; **(3)** retinal plaques were directly linked with Aβ-42 in tears and Aβ-42 was not linked to the expression of dementia symptoms; **(4)** negative test for Aβ-42 in “no AD risk” patient just after eye viral infection suggests that inflammatory conditions do not cause false-positive findings; **(5)** Aβ-42 in tears could have a predictive value in the diagnosis of AD	[110]
Aβ40, Aβ42	CTR *n* = 50 healthy donors (*wide age spectrum 20 to 79*)	Schirmer strips	Electrochemical immunosensor	**(1)** Testing of a new biosensor: relatively high sensitivity and a low detection limit (detection range 1–100 pg/mL); **(2)** Testing of AD classical biomarkers: tears of healthy people were found to contain 10 times more Aβ peptides than their blood (10 pg/mL in tears; 1 pg/mL in blood); Aβ content in healthy subjects was inversely proportional to the age; the youngest age group (ages 20–39) and the oldest age group (ages 60–79) differed significantly in their Aβ ratio (*p* < 0.01).	[111]

CTR—control; SCD—subjective cognitive decline; MCI—mild cognitive impairment; AD—Alzheimer’s dementia; LC-MS/MS—liquid chromatography with tandem mass spectrometry; RP-LC MS/MS—reversed-phase liquid chromatography with tandem mass spectrometry.

### 3.4. Multiple Sclerosis (MS) Disease Tears Biomarkers

Multiple sclerosis (MS) is a chronic autoimmune, and an inflammatory-neurodegenerative disease of the central nervous system, presenting with significant inter- and intraindividual heterogeneity [115,116]. According to biomarkers in body fluids [9,117], some of the mentioned may be found in tears.

One of the most important indicators that helps in the diagnosis of subclinical inflammatory disease of the central nervous system [118,119] and has a predictive and diagnostic value for MS patients with a first suspected symptoms is the detection of oligoclonal bands (OCBs) in cerebrospinal fluid (CSF). OCBs were detectable in the CSF and tears from MS patients [119] but according to other research they are not recommended as biomarkers of disease. Bachhuber [120] analyzed OCB in tear fluid, CSF, and serum samples from 22 patients diagnosed with multiple sclerosis. OCB in tear fluid was not specific for MS but for patients diagnosed with multiple sclerosis or other inflammatory diseases, and it is not recommended that tear OCB detection may be a replacement of CSF OCB detection in MS patients [121].

Using liquid chromatography coupled with tandem mass spectrometry (LC-MS/MS), analysis of tear lipids containing choline, as well as free carnitine, acylcarnitines, and aminoacids, was shown to reflect the pathological conditions of the central nervous system, suggesting their potential biomarker role for MS [5]. Proteomics characterization of released extracellular vesicles (EVs) secreted in CSF and tears of multiple sclerosis patients was presented by Pieragostino et al. [39]. EV has been found to transport the same proteins from the CNS to tears and the CSF, suggesting that EV can become an important diagnostic tool that can be collected in a minimally invasive way.

Interesting research was present by Örnek et al. [7], where compared corneal sensitivity and tear function for Alzheimer’s disease, multiple sclerosis, Parkinson’s disease, Friedreich’s ataxia (FA), and epilepsy (EP) patients. In this research, it was concluded that neurodegenerative diseases may be associated with reduced corneal sensitivity and abnormal tear function.

Also Belviranli et al. [122] found the physical relationship between the quality/quantity of tears and the link to MS disease. Conjunctival impression cytology (CIC) grades, tear break-up time (TBUT), Schirmer 1 test results, and ocular surface disease index (OSDI) scores were observed with the conclusion that mean CIC grade and OSDI scores were higher in the MS group than in the control group, and other parameters (TBUT, Schirmer 1 test) were higher in the control group.

Table 4 presents the candidate biomarkers found in tears for multiple sclerosis.

## 4. Conclusions

Biomarkers are especially important biological factors that can indicate the pathological state of the body, even before the symptoms of the disease are visible. A reliable disease marker is highly desirable, and the biological material in which the markers can be detected is not less important. In this study, we have analyzed the recent literature on biomarkers, which can be present in tears of patients with AD, PD, and MS. We focused on these three neurodegenerative diseases in which tears are indicated to have a high potential as diagnostic material. Interestingly, recent studies have identified some biomarkers present in tears that can be used for the diagnosis and management of these neurodegenerative diseases.

A-synTotal and α-synOligo, CCL2, lactoferrin, norepinephrine, adrenaline, epinephrine, dopamine, α-2-macroglobulin, proteins involved in immune response, lipid metabolism and oxidative stress, protein deglycase DJ-1, S100 superfamily, peroxiredoxin-6, annexin-A5, and glutathione S-transferase-A1, apolipoprotein superfamily; preamine AIC, cathepsin D, acid ceramidase, transitional endoplasmic reticulum ATP-ase, cytoplasmic dynein-1 heavy chain, tripeptidyl-peptidase 1, CATD, ASAH1, and DYHC1 were shown to be potential biomarkers in PD.

The most frequent in AD were lipocalin-1, lysozyme-C, and lacritin, eIF4E, Aβ38, Aβ40, Aβ42, t-Tau, and p-Tau and for MS they were oligoclonal bands (OCBs), lipids containing choline, free carnitine, acylcarnitines, and amino acids.

This area provides interesting data and new insight to help improving clinical outcomes for patients. It seems that many of the proteins described in this work could already be implemented into clinical practice. However, the current state of knowledge is insufficient to clearly indicate the biomarkers of the diseases discussed here. Many of the presented studies are of a pilot nature. In order for their results to constitute a standard for early and non-invasive diagnosis of PD, AD, and MS, all these analyses should be performed on a much larger study group.

Neurodegenerative diseases develop in the body even a decade before the patient begins to notice the first symptoms. That is why it is so important to search for biomarkers in readily available bioliquids. In the future, this could allow the development of commercial screening tests focused on the rapid diagnosis and the implementation of the treatment of diseases of the nervous system before the patient begins to feel the negative effects of the disease.

## Figures and Tables

**Table 1:** Characteristic of tear analysis methods.

Method of Tears Collection	Method of Identification/ Analysis	References
Schirmer strips	Mass spectrometry	[13,24]
Absorbent materials/sponge	ELISA 2D-electrophoresis	[48,51]
Microcapillary tubes	ELISA Multiplex bead analysis	[53,54,64]
	Mass spectrometry Microarray	[48,50]
Micropipette	SDS-PAGE	[49]
Contact lens/Biosensors	Bioelectrochemical field/electrochemical techniques	[55,60,61,62]
	ELISA	[52]

2D electrophoresis—two-dimensional gel electrophoresis; ELISA—enzyme-linked immunosorbent assay; SDS-PAGE—polyacrylamide gel electrophoresis under denaturing conditions.

**Table 4:** Characteristic of biomarkers in multiple sclerosis.

Biomarker/s	Number of Patients	Method of Tears Collection	Method of Identification/Analysis	Results	References
Corneal sensitivity/tear function	AD *n* = 20, MS *n* = 20, PD *n* = 30, Friedreich ataxia (FA) *n* = 10, epilepsy (EP) *n* = 21	Schirmer test	(1) Central corneal sensitivity was measured using a Cochet–Bonnet esthesiometer. (2) Schirmer’s test score. (3) Tear function tests included tear break-up time (TBUT)	**(1)** Mean corneal sensitivity was significantly reduced in AD, MS, PD, and EP patients, in comparison to CTR; **(2)** mean TBUT level was significantly shorter in patients with AD and MS; **(3)** Mean Schirmer’s 1 test score was significantly lower in EP patients; **(4)** The reduction in mean corneal sensitivity in the AD and PD groups was significantly more than in FA and MS groups. Mean TBUT levels in AD, MS, and PD groups were significantly shorter than in FA and EP groups; **(5)** Mean Schirmer’s test scores in AD and PD groups were significantly lower than in MS, FA, and EP groups.	[7]
Conjunctival impression cytology (CIC) grades; tear break-up time (TBUT), Schirmer 1 test results; ocular surface disease index (OSDI) scores	CTR *n* = 33, MS *n* = 33	Schirmer test	(1) TBUT and Schirmer 1 tests were performed; (2) CIC samples were collected	**(1)** Mean CIC grade was higher in the MS group than in the CTR (1.48 ± 0.71 and 0.39 ± 0.56, respectively; *p* = 0.001). In the MS group, the CIC of the 14 participants (42.4%) was grade 2–3. In CTR, CIC of the only one participant (3.3%) was grade 2, and none of them were grade 3. **(2)** TBUT (8.12 ± 3.16, 13.06 ± 4.23 s in MS and CTR, respectively; *p* = 0.001); **(3)** Schirmer 1 test results (8.45 ± 5.75, 17.36 ± 10.89 mm in MS and CTR, respectively; *p* = 0.001) were lower; **(4)** OSDI score (36.36 ± 19.19, 13.70 ± 15.36 in MS and CTR, respectively; *p* = 0.001) was higher in the MS group.	[122]
Oligoclonal bands (OCBs)	No data	No data	Flow cytometry, nLC-ESI-QTOF-MS/MS	**(1)** MVs form neuronal and microglial origin are detectable in the CSF and tears from MS patients	[119]
Oligoclonal bands (OCBs)	MS *n* = 59	Schirmer strips; flush procedure and plastic capillary tubes	Isoelectric focusing in polyacrylamide gels; Immunoblotting	**(1)** The collection of IgG in tears was most reliable by using Schirmer strips; **(2)** The concordance of OCB in tears and CSF of all investigated MS patients was 39% with a high rate of only marginal pattern in tears; **(3)** Not recommended for tear OCB detection as replacement for CSF OCB detection in MS patients.	[121]
Oligoclonal bands (OCBs)	CTR *n* = 44, MS *n* = 22	Capillary tubes or Schirmer strips	ELISA	**(1)** OCB in tear fluid was not specific for MS; **(2)** The presence of OCB in the tear fluid could not be related to the laboratory and clinical parameters.	[120]
Lipids containing choline; free carnitine; acylcarnitines and amino acids	CTR *n* = 21, MS *n* = 12		LC-MS/MS	**(1)** Tear lipidomics showed 30 phospholipids significantly modulated and many sphingomyelins resulted lower in MS; **(2)** The metabolomics approach carried out in both tears and serum highlighted the diagnostic potential of specific amino acids and acylcarnitines.	[5]

ELISA—enzyme-linked immunosorbent assay; LC-MS/MS—liquid chromatography with tandem mass spectrometry; nLC-ESI-QTOF-MS/MS—nano liquid chromatography with tandem mass spectrometry and with ionization type electrospray.

## Data Availability

Not applicable.
